# Generic Meal Patterns Identified by Latent Class Analysis: Insights from NANS (National Adult Nutrition Survey)

**DOI:** 10.3390/nu10030310

**Published:** 2018-03-06

**Authors:** Irina Uzhova, Clara Woolhead, Claire M. Timon, Aifric O’Sullivan, Lorraine Brennan, José L. Peñalvo, Eileen R. Gibney

**Affiliations:** 1 Fundación Centro Nacional de Investigaciones Cardiovasculares Carlos III (CNIC), 28029 Madrid, Spain; iuzhova@cnic.es; 2 UCD Institute of Food and Health, School of Agriculture and Food Science, University College Dublin, D04 V1W8 Belfield, Ireland; clara.woolhead@ucdconnect.ie (C.W.); claire.timon@ucd.ie (C.M.T.); aifric.osullivan@ucd.ie (A.O.); lorraine.brennan@ucd.ie (L.B.); 3 Friedman School of Nutrition Science and Policy, Tufts University, Boston, MA 02111, USA; jose.penalvo@tufts.edu

**Keywords:** latent class analysis, dietary patterns, generic meals, breakfast, light meal, main meal

## Abstract

Nutritional data reduction methods are widely applied in nutrition epidemiology in order to classify individuals into meaningful groups with similar dietary patterns. To date, none of the existing studies have applied latent class analysis to examine dietary patterns which include meal types consumed throughout a day. We investigated main meal patterns followed on weekend and weekdays, and evaluated their associations with cardio-metabolic biomarkers. The analyses were performed within the NANS (National Adult Nutrition Survey) a cross-sectional national food consumption survey of 1500 nationally representative Irish adults. A total number of seven dietary patterns were identified using latent class analysis. The typical meal pattern followed by the majority of the population was characterized by consumption of cereal or toast for breakfast, skipping or consuming a sandwich for light meal, and meat or fish with potatoes, pasta or vegetables for the main meal. Eating patterns differed on weekends, and those participants who consumed meat and eggs for breakfast instead of breakfast cereal and skipped light meal were more likely to have an unhealthier dietary pattern, a higher diastolic blood pressure, and increased serum ferritin. The application of data reduction techniques to simplify the multifaceted nature of dietary data is a useful approach to derive patterns, which might shed further light on the typical dietary patterns followed by populations.

## 1. Introduction

The concept of dietary intake data reduction applied to dietary data has been widely used to classify individuals within a population into meaningful groups with similar diets [1,2]. These diets can be defined “a priori” by using established dietary indices [3] and fitting the data into pre-defined dietary patterns or “a posteriori” by means of statistical classification techniques such as factor analysis (FA), or principal component analysis (PCA) that will result in data-driven dietary patterns [4]. Adherence to “a priori” and “a-posteriori” defined dietary patterns has been extensively investigated for the associations with varied disease outcomes. For example, Mediterranean dietary pattern measured by adherence to “a priori” defined dietary indices was associated with reduced cardiovascular, cancer, and all-cause mortality [5], while a Western dietary pattern derived by Principal component (PCA) and Factor analysis (FA) is linked to type II diabetes, cancer, and biomarkers of obesity and cardiovascular disease (CVD) risk [6,7,8]. Challenges in accurate determination of dietary patterns still exist, primarily in the need for strategies to reduce the complex multidimensional nutritional data down to an interpretable set of observed patterns.

Latent class analysis (LCA) has been widely used in social and behavioral sciences and also applied to relevant qualitative dietary intake data [9,10], however, few studies have used LCA to characterize diets by classifying individuals into categories (or classes) of similar dietary behaviors. Sotres-Alvarez et al. in their investigations concluded that LCA is a useful approach to classify individuals into exclusive classes based on the similarity in dietary behavior, compared to FA, which could be mainly used identify the combinations of foods typically consumed [11]. Furthermore, since LCA is considered a data driven approach, it may show a more realistic picture of what people eat in daily life and provide interesting insights into dietary behavior [12]. For example, in the work by Padmadas et al. the authors used LCA to derive 5 main dietary patterns prevalent among Indian women revealing a heterogeneity of dietary behaviors across the country which none of the previous studies could detect [12].

To date, none of the existing studies have applied LCA to understand the patterns of meal types consumed throughout a day (for breakfast, lunch, dinner, or snacks). Meal analysis has recently received considerable public health interest following arguments that foods are usually consumed in combination within meals, and studying meals instead of focusing on food groups might have an important contribution towards more effective, meal-based, dietary guidance [13,14,15]. However, challenges still exist with the fact that not every dietary assessment tool provides the information on meals consumed. In order to address this limitation, Woolhead et al. developed a generic meal coding system, which allowed the aggregation of complex population food consumption data into generic meals within eating occasions. Those included breakfast, light meal, main meal, and snacks specific for the population considered, and in doing so reduced the complexity of data, allowing for the application of PCA to derive meal patterns [13]. However, the heterogeneity of diet in the studied population still hindered full capture of generic meal patterns with as many as twelve components (meal patterns) explaining only 29% of the total variance. Thus, further investigation is required, and therefore the aims of the present study were to use the novel approach of generic meal coding and apply LCA to derive meal consumption patterns among the Irish population, to study how meal patterns differ between weekdays and weekends, and to evaluate the association of these patterns with cardio-metabolic biomarkers.

## 2. Materials and Methods

### 2.1. Study Overview

The National Adult Nutrition Survey (NANS) was a cross-sectional national food consumption survey carried out between 2008 and 2010 and collected data on habitual dietary intakes, lifestyle, health related indicators, and attitudes towards food and health among a total of 1500 healthy, free-living adults in Ireland [16]. Male and female participants aged 18 years and over residing in the Republic of Ireland were included in the study. A detailed description of the study design and procedures of data collection are described in detail elsewhere (16). The study protocol was approved by the Human Ethics Research Committee of University College Dublin and the University College Cork Research Ethics Committee of the Cork Teaching Hospitals (ECM 3(p) 04/11/08), and all participants provided written informed consent [16].

### 2.2. Demographic, Anthropometric, and Clinical Variables

Demographic information from NANS was used in this analysis including sex, age (years), marital status, and social class. Lifestyle-related variables included smoking habits, alcohol consumption assessing how often the participant consumed alcoholic drinks, supplement use as well as self-identification of being responsible for groceries or cooking. Anthropometry included measurements of height, weight, waist, and hip circumference measured by researchers during the data collection period and were obtained through standardized procedures [16]. Data on serum blood lipids, serum glucose, and C-reactive protein (CRP) was used. Serum triglycerides were analyzed with colorimetric method (Randox Daytona, Randox Laboratories Ltd., Crumlin, UK), serum total cholesterol was analyzed with cholesterol oxidase-enzymatic endpoint method (Randox Daytona), and HDL-c was analyzed with direct clearance method (Randox Daytona). LDL-c was calculated using Friedewald equation. CRP and serum ferritin were analyzed with immunoturbidimetric immunoassay method (Randox Daytona) [16]. Those participants with serum ferritin > 200 mg/L for men and 150 mg/L for women were considered to have increased serum ferritin levels [17] and hypertension if average blood pressure was greater than 140/90 mmHg [18]. Missing values on body mass index (BMI) (*n* = 146), weight (*n* = 87), waist-to-hip ratio (*n* = 226), body fat (*n* = 181), systolic blood pressure (*n* = 185), diastolic blood pressure (*n* = 185), total cholesterol (*n* = 369), triglycerides (*n* = 370), high-density lipoprotein cholesterol (HDL-c) (*n* = 377), low-density lipoprotein cholesterol (LDL-c) (*n* = 387), serum glucose (*n* = 373), and CRP (*n* = 652) were excluded from the analysis. There were no statistically significant differences in sociodemographic characteristics between those who were excluded from and included in the final analysis.

### 2.3. Dietary Assessment and Generic Meals Determination

Four-day semi-weighed food diaries were used to collect participants’ dietary data [16]. Subjects were asked to record and weigh any food, beverage, or supplements at the time of consumption and provide the description of the food, as well as detailed information regarding the preparation method, manufacturer, and time of consumption. Participants were encouraged to include at least one weekend in their dietary record. Weighed Intake Software Program WISP^©^ (Tinuviel Software, Anglesey, UK) version 3.0 used “The Composition of Foods” to generate nutrient intake data [19,20].

The dietary data collected within NANS contained the information on the particular meal types corresponding to the food ingested. For example, the cereals were consumed as a part of breakfast, the participant would indicate “breakfast” as a meal type. There were 11 original meal types collected: breakfast; light meal as a part of the lunch, light meal as a part of the evening meal; main meal as a part of the lunch, main meal as a part of the evening meal; morning, afternoon, evening, and night snacks; alcohol and non-alcoholic beverages. Those meal types were reduced to 5: breakfast, light meal, main meal, snacks and beverages and only first three were used in the analysis.

The methodology applied to create generic meals, from the dietary intake data of NANS is described in detail in Woodhead et al. [13]. In brief, the dietary data from 4 days record contained nutritional information on 2552 food items, which were further reduced into 20 food groups based on their nutritional profile. For example, 78 cereal food items were assigned to the food group “cereals”, and 21 milk food items were assigned to the group “milks”. The 20 food groups included: breads, cereals, milks, fats, fruits, fruit juice, tea/coffee, sugar/jam, cheese, yoghurt, eggs, nuts, vegetables, rice/potatoes/savouries, meat/fish, confectionery/desserts, alcohol, and beverages. The combination of these food groups consumed by one person at a single eating occasion was identified as individual meal. The examples of the individual meals for breakfast are “cereals and milk’ “bread and juice” or “bread and fruit”, recorded for the analysis as “cereal and toast or cereal or toast”. In particular, within Woolhead et al. paper food group combinations were determined based on estimates of prevalence of common combinations followed by the visual inspection to combine similar groupings if required [13] and the total number of 15 generic breakfast meals, 19 light meals, and 15 main meals were previously used. Before applying the reduction of the initial list of generic meals proposed by Woolhead et al., to the ones used in our final analysis, we attempted to fit all original generic meals into LCA. However, based on the assessment of the model fit no interpretable classes were derived, and as such further aggregation of the generic meals was required and similar to Woolhead et al. approach was applied. For example, for a light meal, if the generic meal contained bread, cheese, or meat/fish it was assigned to the group meat/fish/dairy sandwich (MFD sandwich), if the generic meal contained only bread and meat/fish it was assigned to the group meat/fish sandwich (MF sandwich). Thus, our analyses included 4 generic breakfast meal categories (skipped breakfast, cereal and toast or cereal or toast, cooked breakfast, fruits or fruit juice, other); 6 generic light meal categories (skipped light meal, meat fish of dairy sandwich, dairy sandwich, meat or fish sandwich, soups or salads, rice or potato or pasta, other), and 4 generic main meals (skipped main meal, protein and carbohydrate based, protein based, carbohydrates based main meal) (Tables S1 and S2).

### 2.4. Dietary Pattern Identification by Using Latent Class Analysis

Patterns of generic meal consumption were identified by LCA. Three main domains (meal types), each containing several categories were used as described above (Tables S1–S3). The dietary data collected from all 1500 NANS participants across all 4 days was used, resulting in a total number of 6000 observations being included in the LCA. Number of classes were chosen based on the assessment of the model fit, which was based on the combinations of the following parameters: (1) smaller model fit indices, including the Akaike information criterion (AIC) and Bayesian information criterion (BIC); (2) the Bootstrap Likelihood ratio test (BS-LRT) comparing k classes vs. k − 1 class models; and (3) pattern interpretability [21]. Two-class model was first fit to the data and additional classes (up to ten classes) were added until the optimal number of latent classes was identified. In order to account for weekdays/weekends variation, as we have observed that 1.5% (*N* = 22), 51.4% (*N* = 771), and 47.1% (*N* = 707) participants reported the dietary intake for all 4 weekdays, 3 weekdays/1 weekends, and 2 weekdays/2 weekends, respectively, LCA was performed separately for weekdays and weekends. The total number of 3815 and 2185 dietary records were included in the LCA for weekdays and weekends, respectively. The models which fit the data best according to above mentioned criteria included 4 classes for weekdays and 3 classes for weekends data. Using an inclusive maximum-probability approach, dietary records were assigned to the class based on item response probability, which indicates the chance of a particular meal type to be consumed in the particular Latent Class. Latent Classes were used as the predictors for further analyses.

### 2.5. Determination of Dominant Classes

**Figure 1:**
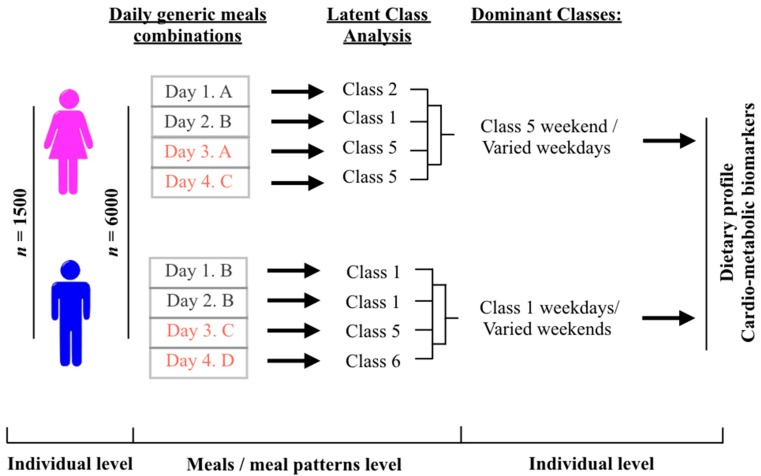
Flow diagram of method to determine meal patterns. The analysis is based on data collected from 1500 male and female participants over 4 days of dietary survey resulting in the total number of 6000 dietary records. A,B,C, and D correspond to the combination of meals types consumed during one day of the dietary survey by a single individual. Day 3 and Day 4 of the dietary survey are weekends. Latent Classes were assigned to each of 6000 dietary records. Separately for weekdays and weekends the dominant Classes were determined based on the adherence to the same latent class over time. Class Varied included the dominant classes representing less than 5% of the total population and the group with no defined dominant classes over 4 days of the survey.

As the Latent Classes were not assigned to the individuals themselves but each day of their dietary record, implying that every individual in our study might fall into different Latent Classes depending on the day of the survey, the reduction from the meal level data (*N* = 6000) back to the individual data (*n* = 1500) was needed in order to study the dietary and phenotypic profile of the meal pattern (Figure 1). Once the Classes were derived for weekdays and weekends, the variables reflecting the Classes which the participant belongs to were pooled together to examine how the Classes differed across all 4 days of record for each participant, and the dominant Classes for each individual were determined. Class was considered dominant if the participant belonged to this Class most of the days (weekdays and weekends classes were treated separately). The group “varied” was used when the weekday/weekend patterns were combined together, and there was no constant meal pattern followed by the participants over 4 days. If the participants adhered to Class 1 at least 2 days on the weekdays, and different Classes during the weekends, they were assigned to the group “Class 1 weekdays/varied”. If the participants adhered to Class 1 at least 2 days on the weekdays and Class 1 at the weekend, they were assigned the group “Class 1 weekdays/Class 1 weekends” etc. Number of participants in each group was identified, and all the groups where the percent of participants didn’t exceed 5% of total population were grouped together with the group “varied”. Variation in clinical cardio-metabolic risk factors was examined across the Dominant Latent Class groups.

### 2.6. Statistical Analysis

The data was normally distributed. Analysis of variance (ANOVA) with Bonferroni post-hoc comparison method was used to compare the daily food, macro- and micronutrients intakes, and clinical variables between the classes of meal patterns. P values were adjusted for age (years), sex (male/female), social class (professional/manager, non-manual skilled, manual skilled, and semi-skilled/unskilled), and energy intake (kcal). Chi-square was used to compare the demographic and lifestyle-related characteristics between the classes of meal patterns. The multivariate adjusted logistic regression analyses were used to study the association between most dominant latent classes and serum ferritin and diastolic blood pressure (DBP). The model was adjusted for age (years), sex (male/female), social class (professional/manager, non-manual skilled, manual skilled, and semi-skilled/unskilled), and energy intake (kcal). LCA was performed using LatentGold 5.1 (Statistical Innovations Inc., Belmont, MA, USA). All other statistical analyses were performed with the Statistical Package for Social Sciences IBM SPSS Statistics for Windows, version 24 (IBM Corp., Armonk, NY, USA). *p* values < 0.05 (two-sided) were considered statistically significant.

## 3. Results

Demographic characteristics of participants are presented in a Table S4. A total number of 3815 and 2185 dietary records represented participants dietary intakes during weekdays and weekends, respectively. Of 3815 weekday dietary records, 60%, 26%, 9%, and 5% fell into Class 1, 2, 3, and 4, respectively (Table 1). The intakes classified as Class 1 had 88% chance to have cereal or toast or both for breakfast, 23% or 28% chance to skip light meal or consume meat or fish (MF) sandwich for light meal and 72% chance to have a protein- and carbohydrate-based main meal. Intakes classified as Class 2 with 65% probability consumed cereal or toast or both for breakfast, a slightly higher chance to have MF sandwich (35%) than meat, fish or dairy (MFD) sandwich (23%) for light meal, and 61% and 23% chance to have a protein- and carbohydrate-based dish for main meal or to skip it, respectively. Intakes at Class 3 were cooked breakfast with a 44% probability, skip light meal with a 57% probability, and a 64% chance to consume a protein- and carbohydrate-based dish for main meal. The intakes that were classified as Class 4 had a 33% probability to consume cereal or toast or both, 27% probability to have fruit or fruit juice for breakfast, a 26% probability to consume soups and salad for light meal, and a 39% chance to have protein and carbohydrates based dish for main meal.

Of 2185 weekend dietary records (Table 1), 57%, 22%, and 21% fell into Class 5, 6, and 7, respectively. Intakes from Class 5 would be 88% more likely to have cereal and toast or cereal or toast for breakfast, 36% more likely to skip light meal, and had 86% probability to have a protein- and carbohydrate-based dish for main meal. Those classified into Class 6 were 70% more likely to have cereal and toast or cereal or toast for breakfast, 24% probability of having MF sandwich for light meal, and protein- and carbohydrate-based meal or just protein-based meal as a main meal with a probability of 39% and 21%, respectively. Class 7 was more likely to be characterized by consumption of cooked breakfast with 45% of probability, skip light meal with 69% of probability, and with 76% probability have protein and carbohydrates based dish for main meal.

Comparing the weekdays Classes between each other, Class 1 presented the highest probability to have cereal or toast or both for breakfast, and protein and carbohydrates based main meal; while Class 2 had zero probability to have fruit or fruit juice for breakfast, and the highest chance to consume meat/fish/dairy (MFD) or meat/fish (MF) sandwich for light meal. Class 3 compared to other 3 classes had the highest probability to have cooked breakfast, and skip light meal. Class 4 had higher probability to have fruits or fruit juice, confectionary, and other foods for breakfast, the highest probability to consume soups or salad as a light meal and carbohydrates based main meal. With respect to weekend classes, Class 5 would have the highest probability to consume cereals or toast or both for breakfast, and protein and carbohydrate based main meal. Class 6 comparing to other 2 Classes had the highest probability to have MFD sandwich or soups and salads for light meal, and protein based main meal; while Class 7 had the highest probability to consume cooked breakfast, and skip light meal.

Results of an overall daily intakes of food groups during weekdays showed that Class 1 was characterized by the highest intakes of breakfast cereals, fruits and fruit dishes, and lowest intakes of eggs and egg dishes. Class 2 was observed to be the highest in grains, rice, pasta, and savories, as well as potatoes and potato dishes. Class 3 was characterized by the highest consumption of soups, sauces and miscellaneous. The consumption of vegetables and vegetable dishes was observed to the lowest for Class 4. As for the overall daily intakes of food groups during weekends, the highest intakes of breakfast cereals, vegetables and vegetable dishes, ice cream and dessert have been observed among Class 5 participants. Highest intakes of grains, rice, pasta, and savories together with nuts and herbs tend to be highest among participants adhering to Class 6, who also were observed to have the lowest consumption meat and potatoes. Those who belongs to Class 7 were characterized by the lowest consumption of breakfast cereal, fruit/fruit dishes, milk and yogurt, as well as bread and rolls (Table 2).

In terms of nutritional quality for weekdays Classes, Class 1 was categorized as the highest in dietary fibre. Class 2 was the lowest on sodium and calcium. Class 3 tend to be the lowest in total energy intake and starch. Regarding the weekends, Class 5 tend to have the highest protein and vitamin C intake and the lowest intakes of Vitamin A. Class 6 had the highest intakes of calcium. Class 7 was observed to have the highest intakes of total fat, including monounsaturated fatty acids (MUFA) as well as the lowest intakes of carbohydrates, starch, sugar, dietary fibre, calcium, and iron (Table 3). Food group intakes within each of the specific meal types (breakfast, light meal, main meal, snack) across the Classes are presented in Tables S5–S8.

Once the participants’ adherence to latent classes for weekends and weekdays were pooled together, 20 groups with the most dominant classes were identified (Table 4). Among all possible combinations, approximately 50% of the sample showed a predominant dietary behavior (dominant class) on weekdays and on weekends, falling into 4 possible combinations with distinctive food intakes (Table 5). Participants who followed Class 1 during weekdays and Class 5 during weekends had significantly (*p* < 0.05) lower intakes of grains, rice and pasta compared to those falling into Class 1 weekdays/Class 6 weekends, and the highest intakes of breakfast cereals compared to the rest of the Classes. They also were observed to have significantly higher intakes of fruits, bread and rolls and lower intakes of meat and meat dishes compared to participants from Class 2 weekdays/Class 5 weekends pattern. In turn, participants from Class 1 weekdays/Class 7 weekends pattern were observed to have the lowest intakes of breakfast cereals among all the Classes and significantly higher intakes of eggs and egg dishes, compared to Class 1 weekdays/Class 5 weekends pattern. As for the Class 2 weekdays/Class 5 weekends pattern, participants who adhered to that Class had the highest intakes of potatoes and potato dishes compared to other three Classes, significantly lower intakes of fruits and higher intakes of meat compared to Class 1 weekdays/Class 5 weekends and Class 1 weekdays/Class 6 weekends.

The analysis of clinical variables with multivariable adjustment across most Dominant Latent Classes (Table 6) showed that participants who followed Class 1 weekdays/Class 7 weekends pattern had significantly (*p* < 0.05) higher DBP compared to those falling into Class 1 weekdays/Class 6 weekends, as well as significantly higher risk of presenting increased serum ferritin (Table S9) comparing to those from Class 1 weekdays/Class 5 weekends pattern (OR: 3.14; 95%CI: 1.63; 6.03).

## 4. Discussion

In this study, we identified the most common meal patterns in Ireland. We used a novel technique, of using generic meal data within LCA, that applied to NANS data allows for identification of food/food group combinations at different eating occasion during the day. Accounting also for difference in patterns between weekdays and weekends, we were able to organize people into meaningful groups with similar dietary behaviour. Thus, a total of 7 distinctive meal patterns were characterized. The majority of Irish adults followed a dietary lifestyle characterised by cereal or toasts for breakfast, skipped or consumed a sandwich as a light meal, and meat or fish with potatoes, pasta or vegetables for the main meal.

A number of methods have been described to elucidate dietary patterns in different populations. Data-driven approaches such as PCA and FA are widely used in nutritional epidemiology [2], whereas LCA has been used mostly in social studies [9] and with limited application to study dietary behaviours. Padmadas et al. applied LCA to understand dietary intake pattern from Indian National Family Health Survey [12]. Seven food groups each with four categories of frequency of intake were used for the analyses, and derived 5 mutually exclusive classes, which reflected the heterogeneity of dietary behaviour among Indian women population. Another study by Sotres-Alvarez et al. also used LCA to derive dietary patterns based on the dietary data from the 3rd cohort of the Pregnancy, Infection and Nutrition (PIN) Study of women [11]. In particular, they used the data from reported intakes of 105 food groups on which individuals were categorized into non-consumers (0 g/day), low consumers and high consumers (below and higher than median), respectively [11]. Three main dietary patterns were derived: Prudent, which was high in fibre, folate, and vitamins; Health Conscious Western—greater intakes of fast food, salty snacks, and sweets, fruits and vegetables; and Hard-Core Western—decreased intakes of fruits and vegetables, nuts and beans, and increased consumption of fried meat, fish, white bread, and sugar sweetened beverages [14]. However, to date most of the studies in the literature that have applied LCA to derive dietary patterns used food-based models which significantly limit the assessment of complexity of the diet, as it only gives the estimation of food types consumed within the identified dietary pattern and does not reflect the timing when the food is consumed or the combination of foods consumed at a single occasion, e.g., breakfast, light meal, or snack. Nor they can be used to explore the sequence of patterns and estimate to what extend a specific type of meal might impact the subsequent food intakes throughout the day. An interesting attempt to circumvent the food based approach and study dietary groups was performed by Wang et al. in the study of Australian men and women [22]. In this particular study, LCA was based on the diets followed such as low-fat, low-fat/low sugar, low-salt, and glycaemic index (GI) diets, prescription diets, gluten-free, vegetarian, vegan, high protein, or lactose free to derived dietary patterns which would determine participants preferences towards these types of diets [22]. The only study, to date, which attempted to examine dietary patterns by meal type was conducted based on Main Meals Repertoire Survey which captured the information on 81 dishes prepared for main meals, for example, beef burger, vegetable soup, fish steak, sausages, roasted lamb, lasagne, etc. without considering side dishes or starters [23].

Although in nutritional epidemiology some studies have attempted to tackle the lack of research involving meal patterns, the application of LCA to study meal intakes remains largely unexplored. The data driven approach using LCA has been utilized previously in Irish adult population in order to determine typical dietary patterns [24], however, it makes it difficult to compare with the meal patterns derived in our study, as the models were food-based and determined only “extreme” patterns: “Healthy”, characterized by high intakes of fruits and vegetables, low fat dairy, and high compliance with DASH dietary index; “Western”-characterised by higher intakes of cereals, breads and potatoes, processed meat and foods from the upper corner of the food pyramid; and “low energy”–included lower intake sweets, red meat, and energy intake overall.

Within the current analysis, meal patterns derived by LCA were determined separately for weekdays and weekends, taking into account the variability that exists in dietary intakes between weekdays and weekends [25]. Alignment of derived meal patterns with actual intake was performed by comparing the actual foods and nutrients intakes across different LCA classes. For example, participants in dietary patterns with highest probability to have breakfast cereal for meal type “breakfast”, bread and rolls for “light meal”, and protein based “main meal” were shown to have the highest actual intake of these food groups. However, there were some discrepancies. The intake of some food items, such as eggs, fish, or meat, which might be consumed as a sandwich, cooked breakfast, or salad, were not in agreement with the actual meat intake when compared. Furthermore, food serving size was not available, and generic meals capture only the types of foods consumed assuming an average portion size [13]. Therefore, there is a possibility that for a group of participants which would consume a small amount of given food but more frequently, this would result in the highest probability of consuming this particular food, but the actual dietary intake would be low. As such, further refinement in generic meal definition including food weight is needed in order to reduce such error.

Linking meal patterns to phenotypes and disease risks, in our study, we observed that meal patterns differed between weekdays and weekends, with results showing that number of participants were more likely to consume cooked breakfast consisted of meat and eggs or roll instead of breakfast cereal on weekends. Those opting to follow this meal pattern on weekends had on average an unhealthier overall diet characterized by lower intakes of vegetables and fruits, milk and dairy, and higher intake of meat and meat dishes, as well as 3.14-fold higher odds for increased serum ferritin. The most studied meal in the scientific literature is breakfast, which shows that breakfast consist of cereals or toast is associated with satiety, overall daily energy intake, and appetite regulation [26,27], as well CVD risk factors [28] and atherosclerosis [29]. The role of other meals, in particular light meal, remained largely unexplored, and requires further research.

There are strengths and limitations to the work presented here. Strengths of this study include the large, nationally representative sample of Irish men and women and the comprehensive nutritional assessment which captured variation in population’s dietary intake over 4 days. The ability to capture overall dietary intake in the identification of our dietary classes by using frequency of consumption instead of incorporating the mean daily food or nutrients intakes in the model, as it has been done for studies using PCA, is another strength. By analysing dietary patterns followed during weekdays and weekends we were able to understand how stable the dietary patterns were, and whether those who follow a particular pattern during the weekdays keep adhering to the same dietary behaviours during weekends. A novel feature of this work was the application of the innovative approach to generate unique generic meals using recently published meal coding system [13,30], that could be translated into other data sets and the use of LCA to examine meal based eating patterns while taking into account meal occasion across the day. The number of classes were identified using standardised criteria which minimized researchers’ involvement into the patterns determination and therefore interpretability of the findings. In the previous study [13] where the meal aggregation method was first developed and applied in principal component analysis in order to identify meal patterns, the main limitation which affected the findings obtained, was a high inter-individual variation in diet across the population. In this work, by reducing the large variation in the meal types, we were able to derive smaller number of meal patterns and categorise all the participants into one of them. On the other hand, a major limitation is the person-centred, data-driven approach which makes the findings non-generalizable to other populations. In addition, within the present study as the population had relatively similar dietary behaviours, and it would be interesting to conduct future analysis with more heterogeneous populations to determine more extreme meal patterns.

## 5. Conclusions

LCA was observed to be a useful exploration tool, which in combination with generic meal-coding system could simplify the complexity of dietary data and derive interpretable meal based dietary patterns. Further work will be directed at refining the generic-meal coding system to include serving size in the meal definition and explore snacking and beverages consumption. These findings could be applied to tackle the chronic diseases by translating the message into public health guidelines and recommendations, complementing current dietary advice to assist the population in achieving the recommended daily intakes of foods and nutrients. Moreover, meal-based dietary guidelines may be easier translate to the population and for the individuals to follow.

## Figures and Tables

**Table 1:** Latent Classes of generic meal intakes.

		Weekdays				Weekends		
Latent Classes		Class 1	Class 2	Class 3	Class 4	Class 5	Class 6	Class 7
*N* (%)		2288 (60.0)	988 (25.9)	360 (9.4)	179 (4.7)	1249 (57.2)	469 (21.5)	467 (21.4)
Meal type	Generic meal	Conditional probabilities ^1^				Conditional probabilities ^1^		
Breakfast	No breakfast	0.044	0.087	0.139	0.030	0.045	0.096	0.153
	Cereal and toast or cereal or toast	0.877	0.648	0.129	0.327	0.883	0.703	0.142
	Cooked breakfast	0.008	0.105	0.444	0.001	0.029	0.056	0.450
	Fruit/fruit juice	0.039	0.000	0.034	0.266	0.005	0.037	0.089
	Other	0.029	0.157	0.251	0.374	0.037	0.106	0.164
Light meal	Skip light meal	0.229	0.011	0.569	0.172	0.364	0.157	0.688
	MFD sandwich	0.113	0.233	0.051	0.038	0.098	0.137	0.067
	Dairy sandwich	0.063	0.080	0.014	0.062	0.068	0.034	0.041
	MF sandwich	0.280	0.348	0.175	0.060	0.204	0.244	0.084
	Soups and salads	0.081	0.008	0.084	0.264	0.057	0.109	0.052
	Rice potato pasta	0.013	0.148	0.000	0.148	0.004	0.101	0.007
	Other	0.220	0.168	0.103	0.253	0.201	0.215	0.059
Main meal	Skip main meal	0.023	0.231	0.049	0.051	0.000	0.169	0.060
	Protein and carbohydrates	0.720	0.607	0.635	0.387	0.857	0.391	0.755
	Protein	0.144	0.150	0.194	0.183	0.105	0.210	0.087
	Carbohydrates	0.081	0.009	0.087	0.216	0.031	0.166	0.069
	Other	0.029	0.000	0.031	0.160	0.005	0.061	0.026

^1^ Values are conditional probabilities to consume one of the listed generic meals given that the participant is classified in one of the following latent classes; *N* (%) is the number of dietary records of 1500 participants over 4 days across all latent classes. MFD—meat/fish/dairy; MF–meat/fish.

**Table 2:** Overall daily intakes ^1^ (g/day or mL/day) of selected food groups by latent classes.

	Weekdays				Weekends		
Latent Classes	Class 1	Class 2	Class 3	Class 4	Class 5	Class 6	Class 7
*N* (%)	2288 (60.0)	988 (25.9)	360 (9.4)	179 (4.7)	1249 (57.2)	469 (21.5)	467 (21.4)
Grains, rice, pasta & savories	55.5 ± 111 ^b,c^	86.6 ± 136 ^a,c,d^	30.8 ± 93.6 ^a,b^	44.1 ± 96.3 ^b^	42.8 ± 93.9 ^f,g^	94.4 ± 163 ^e,g^	62.0 ± 123 ^e,f^
Breakfast cereals	76.4 ± 99.6 ^b,c,d^	45.8 ± 81.3 ^a^	53.7 ± 106 ^a^	39.4 ± 68.9 ^a^	77.2 ± 101 ^f,g^	58.4 ± 88.5 ^e,g^	2.90 ± 16.7 ^e,f^
Potatoes/potato dishes	116 ± 124 ^b,c,d^	163 ± 146 ^a,c,d^	17.3 ± 57.8 ^a,b,d^	47.6 ± 91.2 ^a,b,c^	148 ± 128 ^f^	64.0 ± 121 ^e,g^	140 ± 138 ^f^
Vegetables/vegetable dishes	120 ± 117 ^b,d^	103 ± 113 ^a,d^	116 ± 167 ^d^	70.3 ± 93.5 ^a,b,c^	123 ± 114 ^f,g^	106 ± 141 ^e^	103 ± 113 ^e^
Fruits/fruit dishes	175 ± 191 ^b,c,d^	119 ± 172 ^a^	141 ± 193 ^a^	128 ± 189 ^a^	137 ± 178 ^g^	145 ± 196 ^g^	94.0 ± 170 ^e,f^
Nuts, seeds, herbs	3.0 ± 14.3	2.5 ± 11.9	3.2 ± 13.5	1.0 ± 6.3	2.98 ± 13.0 ^f^	4.85 ± 18.0 ^e,g^	1.51 ± 9.37 ^f^
Milk and yogurt	269 ± 238 ^b,c^	241 ± 246 ^a^	219 ± 252 ^a^	246 ± 240	238 ± 213 ^g^	231 ± 253 ^g^	150 ± 209 ^e,f^
Meat and meat products	176 ± 155 ^d^	191 ± 156 ^c,d^	159 ± 181 ^b^	138 ± 170 ^a,b^	193 ± 144 ^f^	130 ± 154 ^e,g^	204 ± 149 ^f^
Fish/fish dishes	35.0 ± 78.2 ^c^	31.7 ± 78.8 ^a^	22.1 ± 60.6	22.0 ± 56.5	25.3 ± 68.1	20.0 ± 64.8	17.9 ± 58.6
Cheeses	15.0 ± 27.3 ^b,d^	10.4 ± 24.3 ^a,d^	12.3 ± 27.3 ^d^	22.5 ± 32.9 ^a,b,c^	12.7 ± 26.9	15.3 ± 30.3 ^g^	10.7 ± 26.9 ^f^
Eggs/eggs dishes	12.2 ± 36.2 ^b,c,d^	16.7 ± 38.7 ^a,c^	23.2 ± 54.8 ^a,b^	20.4 ± 55.3 ^a^	12.9 ± 35.1 ^f,g^	24.0 ± 53.2 ^e,g^	35.3 ± 50.9 ^e,f^
Biscuits, cakes and pastries	29.9 ± 49.2	26.8 ± 47.9	27.3 ± 48.1	29.5 ± 52.0	32.6 ± 54.0	29.4 ± 59.7	26.8 ± 53.3
Cream, ice-cream and dessert	19.2 ± 55.6	17.7 ± 55.9	13.0 ± 46.4	20.3 ± 61.6	30.8 ± 65.7 ^f,g^	16.6 ± 53.0 ^e^	20.4 ± 56.1 ^e^
Soups and sauces	61.0 ± 117 ^c^	50.8 ± 91.9 ^c^	84.1 ± 151 ^a,b,d^	48.4 ± 108 ^c^	50.4 ± 95.5	53.6 ± 109	48.8 ± 99.2
Breads and rolls	129 ± 83.9 ^b,c^	96.7 ± 84.7 ^a,d^	106 ± 97.2 ^a,d^	147 ± 91.8 ^b,c^	111 ± 78.0 ^g^	119 ± 91.2 ^g^	90.0 ± 82.2 ^e,f^
Sugar, confectionary, snacks	28.6 ± 36.7 ^d^	30.0 ± 39.2	29.5 ± 39.7	37.0 ± 48.0 ^a^	28.3 ± 40.6	33.2 ± 44.9	28.7 ± 40.7
Beverages including water	1421 ± 938	1443 ± 1093	1399 ± 1022	1546 ± 1100	1529 ± 1264 ^g^	1700 ± 1454	1789 ± 1653 ^e^

^1^ Values are mean (±SD). ANOVA with Bonferroni correction was applied. Multiple comparison was performed for Classes 1–4 and Classes 5–7 separately. Classes definition: Weekdays: Class 1–88% cereal or toast for breakfast, 23% skip light meal/28% MF sandwich/22% other for light meal, 72% protein and carbohydrates based for main meal; Class 2–64% cereal or toast for breakfast, 23% MFD sandwich/35% MF sandwich for light meal, 23% skip main meal/60% protein and carbohydrates based for main meal; Class 3–13% cereal or toast/44% cooked breakfast/25% other for breakfast, 57% skip light meal, 64% protein and carbohydrates based for main meal; Class 4–33% cereal and toast/27% fruit/37% other, 26% soups and salads/25 other, 39% protein and carbohydrates based for main meal; ^a^ significant difference from Class 1 (*p* < 0.05); ^b^ significant difference from Class 2 (*p* < 0.001); ^c^ significant difference from Class 3 (*p* < 0.001); ^d^ significant difference from Class 4 (*p* < 0.001). Weekends: Class 5–88% cereal or toast for breakfast, 35% skip light meal/20% MF sandwich/ 20% other for light meal, 85% protein and carbohydrates based for main meal; Class 6–70% cereal or toast for breakfast 24% MF sandwich/22% other for light meal, 39% protein and carbohydrates based for main meal; Class 7–45% cooked breakfast, 69% skip light meal, 76% protein and carbohydrates based for main meal; ^e^ significant difference from Class 5 (*p* < 0.05); ^f^ significant difference from Class 6 (*p* < 0.001); ^g^ significant difference from Class 7 (*p* < 0.001).

**Table 3:** Mean daily macro- and micronutrients intake ^1^ by latent classes.

	Weekdays				Weekends		
Latent Classes	Class 1	Class 2	Class 3	Class 4	Class 5	Class 6	Class 7
*N* (%)	2288 (60.0)	988 (25.9)	360 (9.4)	179 (4.7)	1249 (57.2)	469 (21.5)	467 (21.4)
Energy intake, kcal	1993 ± 749 ^c^	2052 ± 816 ^c^	1678 ± 775 ^a,b,d^	1917 ± 840 ^c^	2074 ± 829	2075 ± 955	2108 ± 1043
Fat, %TE	33.5 ± 8.73 ^c^	34.0 ± 9.66	35.2 ± 12.1 ^a^	34.2 ± 9.20	33.0 ± 9.30 ^g^	33.3 ± 10.3 ^g^	35.6 ± 11.6 ^e,f^
SFA, %TE	13.1 ± 4.65 ^c^	13.2 ± 4.92 ^c^	14.2 ± 6.71 ^a,b^	13.5 ± 4.94	13.0 ± 4.86	13.3 ± 5.41	13.7 ± 5.93
MUFA, %TE	12.0 ± 3.82 ^b^	12.5 ± 4.16 ^a^	12.5 ± 4.97	12.1 ± 3.97	12.0 ± 4.03 ^g^	12.0 ± 4.37 ^g^	13.5 ± 4.78 ^e,f^
PUFA, %TE	6.08 ± 3.54	6.03 ± 3.33	6.42 ± 4.23	6.19 ± 3.17	5.68 ± 2.94 ^g^	5.8 ± 3.55	6.22 ± 3.60 ^e^
Protein, %TE	17.8 ± 5.17 ^c,d^	17.8 ± 5.77 ^c,d^	16.3 ± 5.86 ^a,b^	16.0 ± 5.83 ^a,b^	17.5 ± 5.17 ^f,g^	15.1 ± 5.30 ^e,g^	16.6 ± 5.70 ^e,f^
Carbohydrates, %TE	47.9 ± 9.26 ^b^	46.1 ± 10.1 ^a^	46.6 ± 12.4	47.1 ± 11.9	45.8 ± 9.76 ^g^	46.5 ± 11.9	40.1 ± 12.1 ^e,f^
Starch, g	139 ± 58.0 ^c,d^	138 ± 59.1 ^c^	102 ± 60.8 ^a,b,d^	127 ± 59.5 ^a,c^	136 ± 56.3 ^g^	135 ± 73.0 ^g^	118 ± 61.7 ^e,f^
Sugars, g	92.1 ± 50.0	88.3 ± 55.5	86.0 ± 52.4	89.4 ± 61.8	92.5 ± 55.3 ^g^	93.3 ± 60.1 ^g^	81.7 ± 63.4 ^e,f^
Dietary fiber, g	20.8 ± 9.46 ^b,c,d^	18.3 ± 8.97 ^a,c^	16.4 ± 11.6 ^a,b^	16.2 ± 9.62 ^a,b^	19.6 ± 9.18 ^g^	19.0 ± 11.2 ^g^	15.4 ± 8.74 ^e,f^
Calcium, mg/10 MJ/d	1227 ± 531 ^b^	1070 ± 520 ^a,c,d^	1311 ± 851 ^b^	1247 ± 491 ^b^	1102 ± 496 ^f,g^	1273 ± 639 ^e,g^	944 ± 569 ^e,f^
Iron, mg/10 MJ/d	18.4 ± 22.7	17.9 ± 29	18.8 ± 29.4	16.2 ± 20.7	18.7 ± 22.4 ^g^	19.6 ± 34.7 ^g^	14.7 ± 23.9 ^e,f^
Sodium, mg/10 MJ/d	3072 ± 1113 ^b,c,d^	2837 ± 1164 ^a,c,d^	3420 ± 1535 ^a,b^	3363 ± 1176 ^a,b^	1654 ± 401 ^g^	1617 ± 477	1552 ± 438 ^e^
Vitamin A, mcg/10 MJ/d	1549 ± 2302	1504 ± 4687	1495 ± 1769	1090 ± 1142	2849 ± 1103 ^f,g^	3182 ± 1199 ^e^	3078 ± 1269 ^e^
Vitamin C, mg/10 MJ/d	177 ± 404 ^b^	132 ± 311 ^a^	178 ± 369	115 ± 250	1587 ± 2656 ^f,g^	1211 ± 1387 ^e^	1172 ± 1277 ^e^
Vitamin D, mcg/10 MJ/d	6.45 ± 9.49 ^b^	5.3 ± 8.32 ^a,c^	6.82 ± 10.91 ^b^	4.92 ± 6.41	159 ± 298	149 ± 258	148 ± 355
Vitamin E, mcg/10 MJ/d	16.9 ± 30.3	14.9 ± 35.1	19.4 ± 41.8	17.4 ± 46.5	5.93 ± 9.13	5.11 ± 6.97	4.88 ± 6.86
Folate, mcg/10 MJ/d	491 ± 791 ^c^	441 ± 544 ^c^	508 ± 1082 ^a,b^	408 ± 253	15.7 ± 34.0	18.1 ± 45.1 ^g^	11.8 ± 12.1 ^f^
Vitamin B-12, mcg/10 MJ/d	8.41 ± 28.5	8.32 ± 39.7	7.16 ± 10.8	7.03 ± 13.7	478 ± 467	537 ± 2422	394 ± 435
Vitamin B2, mg/10 MJ per d	3.82 ± 9.77	3.2 ± 7.21	4.12 ± 9.2	4.59 ± 16.1	7.55 ± 15.1	13.3 ± 103	7.19 ± 17.7

^1^ Values are mean (±SD). ANOVA with Bonferroni correction was applied. Multiple comparison was performed for Classes 1–4 and Classes 5–7 separately. Classes definition: Weekdays: Class 1–88% cereal or toast for breakfast, 23% skip light meal/28% MF sandwich/ 22% other for light meal, 72% protein and carbohydrates based for main meal; Class 2–64% cereal or toast for breakfast, 23% MFD sandwich/35% MF sandwich for light meal, 23% skip main meal/60% protein and carbohydrates based for main meal; Class 3–13% cereal or toast/44% cooked breakfast/25% other for breakfast, 57% skip light meal, 64% protein and carbohydrates based for main meal; Class 4–33% cereal and toast/27% fruit/37% other, 26% soups and salads/25 other, 39% protein and carbohydrates based for main meal. a significant difference from Class 1 (*p* < 0.05); ^b^ significant difference from Class 2 (*p* < 0.05); ^c^ significant difference from Class 3 (*p* < 0.05); ^d^ significant difference from Class 4 (*p* < 0.05). Weekends: Class 5–88% cereal or toast for breakfast, 35% skip light meal/20% MF sandwich/20% other for light meal, 85% protein and carbohydrates based for main meal; Class 6–70% cereal or toast for breakfast 24%. MF sandwich/22% other for light meal, 39% protein and carbohydrates based for main meal; Class 7–45% cooked breakfast, 69% skip light meal, 76% protein and carbohydrates based for main meal; ^e^ significant difference from Class 5 (*p* < 0.05); ^f^ significant difference from Class 6 (*p* < 0.05); ^g^ significant difference from Class 7 (*p* < 0.05). SFA—saturated fatty acids; MUFA—monounsaturated fatty acids; PUFA—polyunsaturated fatty acids.

**Table 4:** Identification of dominant classes of NANS participants (*n* = 1500) over 4 days of food diary assessment.

Dominant Class Weekdays	Dominant Class Weekends	*N* (%)
Class 1	-	148 (9.90)
Class 2	-	47 (3.10)
Class 3	-	12 (0.80)
Class 4	-	2 (0.10)
-	Class 5	135 (9.00)
-	Class 6	37 (2.50)
-	Class 7	60 (4.00)
Class 1	Class 5	439 (29.3)
Class 1	Class 6	114 (7.60)
Class 1	Class 7	87 (5.80)
Class 2	Class 5	100 (6.70)
Class 2	Class 6	28 (1.90)
Class 2	Class 7	60 (4.00)
Class 3	Class 5	13 (0.90)
Class 3	Class 6	12 (0.80)
Class 3	Class 7	17 (1.10)
Class 4	Class 5	6 (0.40)
Class 4	Class 6	4 (0.30)
Class 4	Class 7	5 (0.30)
-	-	174 (11.6)

Classes definition: Weekdays: Class 1–88% cereal or toast for breakfast, 23% skip light meal/28% MF sandwich/22% other for light meal, 72% protein and carbohydrates based for main meal; Class 2–64% cereal or toast for breakfast, 23% MFD sandwich/35% MF sandwich for light meal, 23% skip main meal/60% protein and carbohydrates based for main meal; Class 3–13% cereal or toast/44% cooked breakfast/25% other for breakfast, 57% skip light meal, 64% protein and carbohydrates based for main meal; Class 4–33% cereal and toast/27% fruit/37% other, 26% soups and salads/25 other, 39% protein and carbohydrates based for main meal. Weekends: Class 5–88% cereal or toast for breakfast, 35% skip light meal/20% MF sandwich/20% other for light meal, 85% protein and carbohydrates based for main meal; Class 6–70% cereal or toast for breakfast, 24% MF sandwich/22% other for light meal, 39% protein and carbohydrates based for main meal; Class 7–45% cooked breakfast, 69% skip light meal, 76% protein and carbohydrates based for main meal.

**Table 5:** Daily food intakes (g/day) ^1^ by most Dominant Classes.

	Class 1 Weekdays/Class 5 Weekends (*n* = 439)	Class 1 Weekdays/Class 6 Weekends (*n* = 114)	Class 1 Weekdays/Class 7 Weekends (*n* = 87)	Class 2 Weekdays/Class 5 Weekends (*n* = 100)	Class 1 Weekdays (*n* = 148)	Class 5 Weekends (*n* = 135)	Varied (*n* = 477)
Grains, rice, pasta & savories	48.8 ± 57.2 ^g^	67.7 ± 69.9	60.8 ± 72.4	62.7 ± 73.2	64.1 ± 72.7	45.8 ± 58.7^g^	70.3 ± 83.2 ^a,f^
Breakfast cereals	84.1 ± 90.5 ^b,c,d,g^	52.9 ± 67.5 ^a^	41.7 ± 57.4 ^a,e^	51.1 ± 63.8 ^a^	78.9 ± 92.9 ^c,g^	71 ± 80.8^g^	41.7 ± 71.5 ^a,e,f^
Potatoes/potato dishes	133 ± 84.4 ^b,e,g^	93.8 ± 73.4 ^a,d,f^	114 ± 78.8 ^d^	160 ± 90.2 ^b,c,e,g^	102.7 ± 80 ^a,d^	131 ± 87.6 ^b^	110 ± 85.6 ^a,d^
Vegetables/vegetable dishes	124 ± 79.9 ^g^	110 ± 92.2	123 ± 78.3	107 ± 71.7	120 ± 78.5	113 ± 84.3	103 ± 85.8 ^a^
Fruits/fruit dishes	168 ± 147 ^d,g^	168 ± 162 ^d^	144 ± 143	107 ± 131 ^a,b^	158 ± 132.7	139 ± 151	127 ± 143 ^a^
Nuts, seeds, herbs	2.80 ± 9.20	3.50 ± 10.0	3.00 ± 8.50	2.80 ± 10.3	3.13 ± 8.46	2.11 ± 6.65	2.88 ± 9.99
Milk and yogurt	283 ± 206	252 ± 196	215 ± 164	270 ± 257	228 ± 167	235 ± 185	208 ± 184
Meat/meat products	176 ± 90.0 ^d^	152 ± 100 ^d^	175 ± 85.2	209 ± 105 ^a,b^	184 ± 107	176 ± 81.2	181 ± 108
Fish/fish dishes	33.1 ± 39.1 ^a^	30.6 ± 44.2	36.9 ± 42.5	24.3 ± 38.1	27.7 ± 41.2	28.5 ± 40.1	24.1 ± 40.8 ^g^
Cheeses	13.2 ± 17.1	17.5 ± 21.5	11.0 ± 13.7	14.8 ± 22.8	12.7 ± 14.9	12.9 ± 14.5	13.7 ± 18.5
Eggs/eggs dishes	13.3 ± 20.8 ^g^	12.3 ± 18.2 ^g^	20.7 ± 25.1	12.9 ± 18.8 ^g^	19.2 ± 24.3	14.0 ± 20.2	20.8 ± 27.9 ^a,b,d^
Cream, ice-cream and dessert	26.3 ± 39.6 ^g^	18.2 ± 38.1	15.2 ± 25.5	22.2 ± 44.8	18.2 ± 32.5	26.8 ± 43	16.4 ± 29.3 ^a^
Soups and sauces	56.4 ± 66.9	53.8 ± 69.3	65.2 ± 67.4	45.6 ± 59.5	54.7 ± 64.9	59.6 ± 66.1	58.0 ± 73.4
Sugar, confectionary, snacks	29.0 ± 27.4	30.5 ± 26.6	23.4 ± 22.3	30.5 ± 31.8	27.8 ± 26.0	26.6 ± 28.5	32.0 ± 29.5
Breads and rolls	128 ± 62.3 ^d,g^	121 ± 59.2	109 ± 56.8	103 ± 63.0 ^a^	120 ± 57.9	118 ± 66.0	104 ± 63.9 ^a^
Beverages	1393 ± 749 ^g^	1437 ± 1007	1631 ± 809	1499 ± 893	1466 ± 805	1508 ± 841	1600 ± 919 ^a^
Nutritional supplements	59.1 ± 115	36.4 ± 124	69.2 ± 174	56.0 ± 111	52.9 ± 108	48.7 ± 112	50.0 ± 118
Biscuits and cakes	33.8 ± 36.5	25.8 ± 35.5	31.0 ± 28.7	24.0 ± 29.7	29.6 ± 33.6	29.1 ± 31.3	27.6 ± 36.5
Butter and oils	18.0 ± 17.7	14.8 ± 18.3	14.3 ± 12.2	15.0 ± 13.8	14.8 ± 18.3	15.2 ± 16.4	13.3 ± 14.7

^1^ Values are mean (±SD). ANOVA with Bonferroni correction was applied; ^a^ significant difference from Class 5 weekends and Class 1 weekdays (*p* < 0.05); ^b^ significant difference from Class 6 weekends and Class 1 weekdays (*p* < 0.05); ^c^ significant difference from Class 7 weekends and Class 1 weekdays; ^d^ significant difference from Class 5 weekends and Class 2 weekdays (*p* < 0.05); ^e^ significant difference from Class 1 weekdays; ^f^ significant difference from Class 5 weekends; ^g^ significant difference from Class “Varied”. Classes definition: Weekdays: Class 1–88% cereal or toast for breakfast, 23% skip light meal/28% MF sandwich/22% other for light meal, 72% protein and carbohydrates based for main meal; Class 2–64% cereal or toast for breakfast, 23% MFD sandwich/35% MF sandwich for light meal, 23% skip main meal/60% protein and carbohydrates based for main meal. Weekends: Class 5–88% cereal or toast for breakfast, 35% skip light meal/20% MF sandwich/20% other for light meal, 85% protein and carbohydrates based for main meal; Class 6–70% cereal or toast for breakfast, 24% MF sandwich/22% other for light meal, 39% protein and carbohydrates based for main meal; Class 7–45% cooked breakfast, 69% skip light meal, 76% protein and carbohydrates based for main meal. Varied–included the dominant classes representing less than 5% of the total population and the group with no defined dominant classes over 4 days of the record.

**Table 6:** Clinical variables by most dominant latent classes computed over 4 days records (weekdays and weekends).

	Class 1 Weekdays/Class 5 Weekends (*n* = 439)	Class 1 Weekdays/Class 6 Weekends (*n* = 114)	Class 1 Weekdays/Class 7 Weekends (*n* = 87)	Class 2 Weekdays/Class 5 Weekends (*n* = 100)	Class 1 Weekdays (*n* = 148)	Class 5 Weekends (*n* = 153)	Varied (*n* = 459)
BMI, kg/m^2^	26.9 ± 4.71	27.0 ± 6.03	27.3 ± 5.04	27.2 ± 4.98	27.4 ± 4.83	27.2 ± 4.39	27.0 ± 5.13
Weight, kg	76.4 ± 14.8	76.8 ± 17.6	77.5 ± 16.8	78.7 ± 16.2	78.8 ± 17.3	78.9 ± 15.4	77.8 ± 16.6
Waist-to-hip ratio	0.88 ± 0.08	0.86 ± 0.08	0.87 ± 0.09	0.89 ± 0.09	0.87 ± 0.08	0.89 ± 0.09	0.88 ± 0.09
Body fat, %	29.7 ± 8.81	28.6 ± 9.55	30.1 ± 7.79	29.0 ± 9.26	28.9 ± 9.05	29.6 ± 8.25	28.7 ± 9.51
SBP, mmHg	126 ± 18.4	122 ± 15.4	125 ± 15.8	125 ± 17.2	127.9 ± 19	124.2 ± 17.4	123.8 ± 17.9
DBP, mmHg	78.2 ± 10.7	75.6 ± 9.51 ^c^	79.6 ± 10.5 ^b^	78.3 ± 9.94	79.3 ± 11.5	77.4 ± 11	78.2 ± 10.7
Total cholesterol, mmol/L	4.99 ± 1.07	4.91 ± 0.83	4.89 ± 0.98	5.16 ± 0.94	4.81 ± 1.1	4.89 ± 0.94	4.91 ± 0.98
Triglycerides, mmol/L	1.31 ± 0.79	1.26 ± 0.68	1.26 ± 0.77	1.47 ± 0.94	1.26 ± 0.84	1.37 ± 0.77	1.29 ± 0.74
HDL-c, mmol/L	1.58 ± 0.43	1.53 ± 0.41	1.60 ± 0.5	1.57 ± 0.37	1.51 ± 0.4	1.5 ± 0.36	1.57 ± 0.46
LDL-c, mmol/L	2.81 ± 0.90	2.81 ± 0.74	2.72 ± 0.85	2.96 ± 0.87	2.73 ± 0.9	2.77 ± 0.84	2.75 ± 0.85
Glucose, mmol/L	5.42 ± 1.17	5.17 ± 0.85	5.50 ± 1.63	5.41 ± 0.96	5.23 ± 0.89	5.33 ± 0.89	5.26 ± 1.29
CRP, mg/L	2.72 ± 2.82	2.6 ± 2.57	3.13 ± 2.96	3.16 ± 4.23	2.46 ± 2.73	2.77 ± 2.53	2.66 ± 2.93
Serum ferritin, ng/mL	121 ± 117 ^c^	105 ± 100	148 ± 152 ^a^	144 ± 130	123 ± 123.4	112 ± 97.9	109 ± 94.9

^1^ Values are mean and standard deviation. *P* values are adjusted for age, gender, social class, and energy intake. ^a^ significant difference from Class 5 weekends and Class 1 weekdays (*p* < 0.05); ^b^ significant difference from Class 6 weekends and Class 1 weekdays (*p* < 0.05); ^c^ significant difference from Class 7 weekends and Class 1 weekdays. Classes definition: Weekdays: Class 1–88% cereal or toast for breakfast, 23% skip light meal/28% MF sandwich/22% other for light meal, 72% protein and carbohydrates based for main meal; Class 2–64% cereal or toast for breakfast, 23% MFD sandwich/35% MF sandwich for light meal, 23% skip main meal/60% protein and carbohydrates based for main meal. Weekends: Class 5–88% cereal or toast for breakfast, 35% skip light meal/20% MF sandwich/20% other for light meal, 85% protein and carbohydrates based for main meal; Class 6–70% cereal or toast for breakfast, 24% MF sandwich/22% other for light meal, 39% protein and carbohydrates based for main meal; Class 7–45% cooked breakfast, 69% skip light meal, 76% protein and carbohydrates based for main meal. Varied–included the dominant classes representing less than 5% of the total population and the group with no defined dominant classes over 4 days of the record.

## Supplementary Materials

The following are available online at http://www.mdpi.com/2072-6643/10/3/310/s1, Table S1: Recoding of generic meal codes and descriptions for the meal type “breakfast”, Table S2: Recoding of generic meal codes and descriptions for the meal type “light meal”, Table S3: Recoding of generic meal codes and descriptions for the meal type “main meal”, Table S4: Demographic and lifestyle-related characteristics of National Adult Nutrition Survey (NANS) population presented by gender, Table S5: Intakes (g/day or mL/day) of selected foods by latent classes during breakfast, Table S6: Intakes (g/day or mL/day) of selected foods by latent classes during light meal, Table S7: Intakes (g/day or mL/day) of selected foods by latent classes during main meal, Table S8: Intakes (g/day or mL/day) of selected foods by latent classes during snacking occasions, Table S9: Association between most dominant latent classes computed over 4 days records (weekdays and weekdays) and serum ferritin and DBP.
