# Supplementary material for: Generic Meal Patterns Identified by Latent Class Analysis: Insights from NANS (National Adult Nutrition Survey)
Source: Nutrients. 2018 Mar 6;10(3):310. doi: 10.3390/nu10030310 (PMC5872728; doi:10.3390/nu10030310)
Supplement: Supplementary file 1 [file nutrients-10-00310-s001.docx]

**Supplemental Material**

**Supplemental Table 1.** Recoding of generic meal codes and descriptions for the meal type “breakfast”

| New Generic Meal Code and Description | | Consumption Frequency,  *n* (%) | Previous Generic Meal Code and Description | | Consumption Frequency,  *n* (%) |
| --- | --- | --- | --- | --- | --- |
| **0** | Skip breakfast | 412 (6.9) | **0** | No breakfast | 412 (6.9) |
| **1** | Cereal and toast or cereal or toast | 4287 (71.5) | **1.1** | Cereals and milk and bread and  juice | 330 (5.5) |
|  |  |  | **1.2** | Cereals and milk and bread and fruit | 147 (2.5) |
|  |  |  | **1.3** | Cereals and milk and bread and other | 559 (9.3) |
|  |  |  | **2.1** | Cereals and milk and juice | 480 (8.0) |
|  |  |  | **2.2** | Cereals and milk and fruit | 305 (5.1) |
|  |  |  | **2.3** | Cereals and milk and other | 1383 (23.1) |
|  |  |  | **3.3** | Bread and fruit | 170 (2.8) |
|  |  |  | **3.4** | Bread and juice | 88 (1.5) |
|  |  |  | **3.5** | Bread and other | 825 (13.8) |
| **2** | Cooked breakfast | 549 (9.2) | **3.1** | Bread and cooked breakfast | 359 (6.0) |
|  |  |  | **3.2** | Bread and egg | 190 (3.2) |
| **3** | Fruit/fruit juice | 236 (3.9) | **4.2** | Fruit/juice | 236 (3.9) |
| **4** | Other | 516 (8.6) | **4.1** | Confectionery | 265 (4.4) |
|  |  |  | **4.3** | Other | 251 (4.2) |
|  | Total | 6000 (100) |  | Total | 6000 (100) |

**Supplemental Table 2.** Recoding of generic meal codes and descriptions for the meal type “light meal”.

| New Generic Meal Code and Description | | Consumption Frequency,  *n* (%) | Previous Generic Meal Code and Description | | Consumption Frequency,  *n* (%) |
| --- | --- | --- | --- | --- | --- |
| **0** | Skip light meal | 1722 (28.7) | **0** | Skip light meal | 1722 (28.7) |
| **1** | MFD sandwich | 678 (11.3) | **1.1** | Bread cheese meat/fish vegetables | 177 (3.0) |
|  |  |  | **1.2** | Bread cheese meat/fish fat | 185 (3.1) |
|  |  |  | **1.3** | Bread cheese meat/fish fat vegetables | 150 (2.5) |
|  |  |  | **1.4** | Bread cheese meat/fish and other | 166 (2.8) |
| **2** | Dairy sandwich | 343 (5.7) | **2.1** | Bread cheese vegetables | 72 (1.2) |
|  |  |  | **2.2** | Bread cheese fat | 120 (2.0) |
|  |  |  | **2.3** | Bread cheese vegetables fat | 81 (1.4) |
|  |  |  | **2.4** | Bread cheese and other | 70 (1.2) |
| **3** | MF sandwich | 1438 (24.0) | **3.1** | Bread meat/fish vegetables | 351 (5.9) |
|  |  |  | **3.2** | Bread meat/fish fat | 491 (8.2) |
|  |  |  | **3.3** | Bread meat/fish vegetables fat | 309 (5.2) |
|  |  |  | **3.4** | Bread meat/fish and other | 287 (4.8) |
| **4** | Soup and salad | 460 (7.7) | **4** | Soups | 342 (5.7) |
|  |  |  | **5** | Salads | 118 (2.0) |
| **5** | Rice potato pasta | 207 (3.5) | **8** | Rice potato pasta | 207 (3.5) |
| **6** | Other | 1162 (19.2) | **6**  **7**  **9** | Egg based  Bread and other  Other | 231 (3.9) |
|  |  |  |  |  | 380 (6.3) |
|  |  |  |  |  | 541 (9.0) |
|  | Total | 6000 (100) |  | Total | 6000 (100) |

MFD – meat/fish/dairy

**Supplemental Table 3.** Recoding of generic meal codes and descriptions for the meal type “main meal”.

| New Generic Meal Code and Description | | Consumption Frequency,  *n* (%) | Previous Generic Meal Code and Description | | Consumption Frequency,  *n* (%) |
| --- | --- | --- | --- | --- | --- |
| **0** | Skip main meal | 347 (5.8) | **0** | Skip main meal | 347 (5.8) |
| **1** | Protein and carbohydrates | 4130 (68.8) | **1.1** | Meat/fish and rice/potato/pasta and vegetable and soups/sauces and confectionery | 399 (6.7) |
|  |  |  | **1.3** | Meat/fish and rice/potato/pasta and vegetable and confectionery | 519 (8.7) |
|  |  |  | **2.1** | Meat/fish and rice/potato/pasta and soups/sauces and confectionery | 283 (4.7) |
|  |  |  | **1.2** | Meat/fish and rice/potato/pasta and vegetable and soups/sauces | 779 (13.0 |
|  |  |  | **1.4** | Meat/fish and rice/potato/pasta and vegetable and other | 1247 (20.8) |
|  |  |  | **2.2** | Meat/fish and rice/potato/pasta and soups/sauces | 243 (4.1) |
|  |  |  | **2.3** | Meat/fish and rice/potato/pasta and other | 660 (11.0) |
| **2** | Protein | 874 (14.6) | **3.1** | Meat/fish and vegetable and confectionery | 153 (2.6) |
|  |  |  | **5** | Meat/fish and confectionery | 145 (2.4) |
|  |  |  | **3.2** | Meat/fish and vegetable | 287 (4.8) |
|  |  |  | **6** | Meat/fish no vegetables and other | 289 (4.8) |
| **3** | Carbohydrates | 472 (7.9) | **4** | Rice/potato/pasta and vegetable | 218 (3.6) |
|  |  |  | **7** | Rice/potato/pasta and no vegetables | 254 (4.2) |
| **4** | Other | 177 (3.0) | **8** | Other | 177 (3.0) |
|  | Total | 6000 (100) |  | Total | 6000 (100) |

**Supplemental Table 4.** Demographic and lifestyle-related characteristics of National Adult Nutrition Survey (NANS) population presented by gender.

|  | Male  (*n*=740) | Female  (*n*=760) | Total  (*n*=1500) |
| --- | --- | --- | --- |
| **Age, years** | 43.8±17.2 | 45.2±16.8 | 44.5±17.0 |
| **Energy intake** | 2237±663 | 1698±471 | 2013±656 |
| **Social class** |  |  |  |
| Professional/manager | 322 (44.5) | 348 (48.9) * | 670 (46.7) |
| Non-manual skilled | 113 (15.6) | 154 (21.7) | 267 (18.6) |
| Manual skilled | 131 (18.1) | 82 (11.5) | 213 (14.8) |
| Semi-skilled /unskilled | 158 (21.8) | 127 (17.9) | 285 (19.9) |
| **Supplement use** |  |  |  |
| Yes | 194 (26.5) | 268 (35.8) * | 462 (31.2) |
| No | 439 (60.1) | 363 (48.5) | 802 (54.2) |
| Sometimes | 98 (13.4) | 118 (15.8) | 216 (14.6) |
| **Smoking** |  |  |  |
| Current smoker | 147 (20.2) | 156 (20.8) | 303 (20.5) |
| Former smoker | 202 (27.7) | 302 (27.0) | 405 (27.4) |
| Never smoker | 380 (52.1) | 392 (52.2) | 722 (52.2) |
| **Alcohol consumption** |  |  |  |
| Almost every day | 322 (44.1) | 237 (31.6) * | 559 (37.8) |
| Once a week | 170 (23.3) | 170 (22.7) | 340 (23.0) |
| 2 times per / month | 64 (8.80) | 86 (11.5) | 150 (10.1) |
| Once a month or less | 99 (13.6) | 131 (17.5) | 230 (15.5) |
| Never | 75 (10.3) | 126 (16.8) | 201 (13.6) |
| **Responsible for grocery** |  |  |  |
| Myself | 211 (28.5) | 555 (73.0) * | 766 (51.1) |
| Others | 529 (71.5) | 205 (27.0) | 734 (48.9) |
| **Responsible for cooking** |  |  |  |
| Myself | 239 (32.3) | 585 (77.0) * | 824 (54.9) |
| Others | 501 (67.7) | 175 (23.0) | 676 (45.1) |

Values are presented as N (%) or mean (±SD). * significant difference between males and females (chi-square analysis is performed).

**Supplemental Table 5.** Intakes^1^ (g/day or ml/day) of selected foods by latent classes during breakfast.

|  | Weekdays | | | | |  | Weekends | | |
| --- | --- | --- | --- | --- | --- | --- | --- | --- | --- |
| **Latent Classes** | **Class 1** | **Class 2** | **Class 3** | **Class 4** | |  | **Class 5** | **Class 6** | **Class 7** |
| **N (%)** | **2288 (60.0)** | **988 (25.9)** | **360 (9.4)** | **179 (4.7)** | |  | **1249 (57.2)** | **469 (21.5)** | **467 (21.4)** |
| Breakfast cereals | 72.5±96.8 ^bcd^ | 41.3±77.4 ^a^ | 45.9±97.2 ^a^ | | 34.1±61.1 ^a^ | | 73.5±98.2 ^fg^ | 55.6±86.5 ^eg^ | 0.00±0.00 ^ef^ |
| Fruits/fruit dishes | 64.6±107 ^bd^ | 45.8±99.6 ^a^ | 59.1±107 ^d^ | | 32.3±82.6 ^ac^ | | 51.8±94.5 | 63.4±120 | 51.1±113 |
| Milk and yogurt | 112±111 ^bc^ | 97.4±115 ^ac^ | 78.0±108 ^ab^ | | 95.0±121 | | 111±110 ^g^ | 104±115 ^g^ | 43.9±80.2 ^ef^ |
| Meat/meat products | 0.37±5.65 ^bcd^ | 11.2±35.1^ac^ | 7.39±27.1 ^ab^ | | 10.7±32.9 ^a^ | | 1.93±21.8 ^g^ | 6.10±25.7 ^g^ | 41.4±65.2 ^ef^ |
| Eggs/eggs dishes | 0.85±9.63 ^bcd^ | 12.3±32.4 ^ac^ | 8.18±25.7 ^ab^ | | 9.89±34.2 ^a^ | | 2.18±13.5 ^fg^ | 6.85±27.2 ^eg^ | 29.4±44.5 ^ef^ |
| Sugar, confectionary, snacks | 6.21±11.0 | 6.69±12.8 | 5.01±9.62 | | 6.02±10.8 | | 7.02±11.9 ^g^ | 5.84±10.5 | 4.24±11.0 ^e^ |
| Breads and rolls | 26.7±37.1 ^b^ | 40.3±48.2 ^acd^ | 30.5±42.0 ^b^ | | 26.0±39.1 ^b^ | | 34.5±39.8 ^g^ | 30.5±41.7 ^g^ | 42.6±47.8 ^ef^ |
| Beverages (incl. water) | 251±233 | 263±246 | 243±246 | | 242±216 | | 263±241 | 256±258 | 278±317 |

^1^ Values are mean (±SD). ANOVA with Bonferroni correction was applied. Multiple comparison was performed for Classes 1-4 and Classes 5-7 separately. Classes definition: Weekdays: Class 1—88% cereal or toast for breakfast, 23% skip light meal / 28% MF sandwich/ 22% other for light meal, 72% protein and carbohydrates based for main meal; Class 2—64% cereal or toast for breakfast, 23% MFD sandwich / 35% MF sandwich for light meal, 23% skip main meal / 60% protein and carbohydrates based for main meal; Class 3—13% cereal or toast / 44% cooked breakfast / 25% other for breakfast, 57% skip light meal, 64% protein and carbohydrates based for main meal; Class 4—33% cereal and toast / 27% fruit / 37% other, 26% soups and salads / 25 other, 39% protein and carbohydrates based for main meal. ^a^ significant difference from Class 1 (*p* < 0.05); ^b^ significant difference from Class 2 (*p* < 0.001); ^c^ significant difference from Class 3 (*p* < 0.001); ^d^ significant difference from Class 4 (*p* < 0.001). Weekends: Class 5—88% cereal or toast for breakfast, 35% skip light meal / 20% MF sandwich/ 20% other for light meal, 85% protein and carbohydrates based for main meal; Class 6—70% cereal or toast for breakfast 24% MF sandwich / 22% other for light meal, 39% protein and carbohydrates based for main meal; Class 7—45% cooked breakfast, 69% skip light meal, 76% protein and carbohydrates based for main meal. ^e^ significant difference from Class 5 (*p* < 0.05); ^f^ significant difference from Class 6 (*p* < 0.001); ^g^ significant difference from Class 7 (*p* < 0.001).

**Supplemental Table 6.** Intakes^1^ (g/day or ml/day) of selected foods by latent classes during light meal.

|  | Weekdays | | | |  | Weekends | | |
| --- | --- | --- | --- | --- | --- | --- | --- | --- |
| **Latent Classes** | **Class 1** | **Class 2** | **Class 3** | **Class 4** |  | **Class 5** | **Class 6** | **Class 7** |
| ***N* (%)** | **2288 (60.0)** | **988 (25.9)** | **360 (9.4)** | **179 (4.7)** |  | **1249 (57.2)** | **469 (21.5)** | **467 (21.4)** |
| Grains, rice, pasta & savouries | 2.16±18.9 ^bcd^ | 9.5±46.2 ^ad^ | 13.0±57.5 ^ad^ | 28.3±84.3 ^abc^ |  | 2.12±22.5 ^f^ | 24.2±79.0 ^eg^ | 1.62±22.3 ^f^ |
| Potatoes/potato dishes | 3.26±22.6 ^bcd^ | 9.62±45.0 ^ad^ | 9.06±41.2 ^ad^ | 33.5±76.5 ^abc^ |  | 3.01±21.8 ^f^ | 21.3±65.6 ^eg^ | 1.43±13.1 ^f^ |
| Vegetables/vegetable dishes | 33.7±60.6 ^bcd^ | 8.08±30.3 ^acd^ | 22.4±79.2 ^abd^ | 47.2±64.4 ^abc^ |  | 20.5±54.5 ^fg^ | 36.7±73.3 ^eg^ | 8.34±33.4 ^ef^ |
| Fruits/fruit dishes | 41.4±88.7 ^bc^ | 7.19±36.4 ^ad^ | 6.97±33.7 ^ad^ | 34.0±99.3 ^bc^ |  | 21.0±68.3 ^g^ | 24.6±73.7 ^e^ | 6.92±34.9 ^ef^ |
| Milk and yogurt | 8.11±24.3 ^bc^ | 0.90±7.99 ^ad^ | 2.59±13.9 ^ad^ | 6.67±25.4 ^bc^ |  | 31.2±69.2 ^g^ | 36.7±74.8 ^e^ | 9.97±46.2 ^ef^ |
| Meat and meat products | 34.5±58.5 ^bcd^ | 13.6±45.6 ^ad^ | 10.3±43.1 ^ad^ | 59.5±69.3 ^abc^ |  | 22.6±53.3 ^fg^ | 53.0±87.7 ^eg^ | 7.25±27.7 ^ef^ |
| Fish and fish dishes | 8.84±33.5 ^bc^ | 2.92±19.0 ^ad^ | 4.28±24.8 ^ad^ | 11.6±37.1 ^bc^ |  | 22.6±53.3 ^f^ | 53.0±87.7 ^eg^ | 7.25±27.7 ^f^ |
| Cheeses | 10.8±22.0 ^bcd^ | 2.32±10.4 ^ad^ | 1.23±8.60 ^ad^ | 15.4±26.2 ^abc^ |  | 7.23±19.3 ^g^ | 8.19±20.0 ^g^ | 3.66±12.6 ^ef^ |
| Eggs and eggs dishes | 7.17±27.0 ^bc^ | 0.89±9.00 ^ad^ | 1.52±11.8 ^a^ | 6.91±32.8 ^b^ |  | 7.18±27.9 ^g^ | 9.16±30.1 ^g^ | 1.50±13.2 ^ef^ |
| Biscuits, cakes and pastries | 8.11±24.3 ^bc^ | 0.90±7.99 ^ad^ | 2.59±13.9 ^a^ | 6.67±25.4 ^b^ |  | 6.49±22.3 ^g^ | 8.71±34.9 ^g^ | 2.75±16.9 ^ef^ |
| Cream, ice-cream and dessert | 2.31±22.2 ^d^ | 0.62±10.2 ^d^ | 0.93±15.7 ^d^ | 7.63±38.1 ^abc^ |  | 2.46±18.8 | 4.42±23.3 ^g^ | 0.53±7.68 ^f^ |
| Soups and sauces | 31.7±90.4 ^b^ | 13.1±59.6 ^acd^ | 42.1±105 ^b^ | 43.0±102 ^b^ |  | 17.3±64.2 ^f^ | 30.4±86.6 ^eg^ | 14.2±63.8 ^f^ |
| Sugar, confectionary, snacks | 6.22±14.69 ^bc^ | 2.22±9.59 ^ad^ | 1.92±8.43 ^ad^ | 7.27±16.2 ^bc^ |  | 3.90±12.5 ^f^ | 6.66±22.2 ^eg^ | 2.16±12.2 ^f^ |
| Breads and rolls | 73.3±50.1 ^bcd^ | 15.4±37.1 ^ad^ | 12.5±36.6 ^ad^ | 82.7±52.9 ^abc^ |  | 46.3±50.9 ^fg^ | 53.6±58.1 ^eg^ | 16.5±39.1 ^ef^ |
| Beverages (incl. water) | 305±285 ^bcd^ | 87.7±196 ^ad^ | 124±252 ^ad^ | 361±270 ^abc^ |  | 189±251 ^fg^ | 324±355 ^eg^ | 77.5±201 ^ef^ |

^1^ Values are mean (±SD). ANOVA with Bonferroni correction was applied. Multiple comparison was performed for Classes 1-4 and Classes 5-7 separately. Classes definition: Weekdays: Class 1—88% cereal or toast for breakfast, 23% skip light meal / 28% MF sandwich/ 22% other for light meal, 72% protein and carbohydrates based for main meal; Class 2—64% cereal or toast for breakfast, 23% MFD sandwich / 35% MF sandwich for light meal, 23% skip main meal / 60% protein and carbohydrates based for main meal; Class 3—13% cereal or toast / 44% cooked breakfast / 25% other for breakfast, 57% skip light meal, 64% protein and carbohydrates based for main meal; Class 4—33% cereal and toast / 27% fruit / 37% other, 26% soups and salads / 25 other, 39% protein and carbohydrates based for main meal. ^a^ significant difference from Class 1 (*p* < 0.05); ^b^ significant difference from Class 2 (*p* < 0.001); ^c^ significant difference from Class 3 (*p* < 0.001); ^d^ significant difference from Class 4 (*p* < 0.001). Weekends: Class 5—88% cereal or toast for breakfast, 35% skip light meal / 20% MF sandwich/ 20% other for light meal, 85% protein and carbohydrates based for main meal; Class 6—70% cereal or toast for breakfast 24% MF sandwich / 22% other for light meal, 39% protein and carbohydrates based for main meal; Class 7—45% cooked breakfast, 69% skip light meal, 76% protein and carbohydrates based for main meal. ^e^ significant difference from Class 5 (*p* < 0.05); ^f^ significant difference from Class 6 (*p* < 0.001); ^g^ significant difference from Class 7 *p* < 0.001).

**Supplemental Table 7.** Intakes^1^ (g/day or ml/day) of selected foods by latent classes during main meal.

|  | Weekdays | | | | | Weekends | | |
| --- | --- | --- | --- | --- | --- | --- | --- | --- |
| **Latent Classes** | **Class 1** | | **Class 2** | **Class 3** | **Class 4** | **Class 5** | **Class 6** | **Class 7** |
| ***N* (%)** | **2288 (60.0)** | | **988 (25.9)** | **360 (9.4)** | **179 (4.7)** | **1249 (57.2)** | **469 (21.5)** | **467 (21.4)** |
| Grains, rice, pasta & savouries | | 51.1±105 ^bcd^ | 72.2±124 ^acd^ | 11.8±55.8 ^ab^ | 0.00±0.00 ^ab^ | 37.7±84.5 ^fg^ | 63.0±139 ^e^ | 54.0±117 ^e^ |
| Potatoes/potato dishes | | 112±121 ^bcd^ | 150±134 ^acd^ | 2.00±20.8 ^ab^ | 0.00±0.00 ^ab^ | 141±120 ^fg^ | 34.1±84.9 ^eg^ | 126±121 ^ef^ |
| Vegetables/vegetable dishes | | 84.0±96.1 ^d^ | 88.1±104 ^d^ | 80.6±134 ^d^ | 10.7±49.0 ^abc^ | 98.4±92.8 ^fg^ | 64.2±120 ^eg^ | 80.5±96.0 ^ef^ |
| Fruits/fruit dishes | | 21.1±66.2 ^d^ | 18.0±69.3 ^d^ | 21.7±73.1 ^d^ | 1.30±10.0 ^abc^ | 20.8±70.5 ^g^ | 15.0±59 | 10.0±49.0 ^e^ |
| Milk and yogurt | | 51.7±117 ^d^ | 57.2±131 ^cd^ | 36.9±95.6 ^bd^ | 7.80±49.2 ^abc^ | 44.6±114 ^f^ | 27.2±81.2 ^e^ | 43.9±122 |
| Meat and meat products | | 135±135 ^bd^ | 155±138 ^acd^ | 127±170 ^bd^ | 51.0±134 ^abc^ | 160±125 ^fg^ | 59.0±115 ^eg^ | 142±123 ^ef^ |
| Fish and fish dishes | | 25.3±68.3 ^d^ | 26.4±73.2 ^d^ | 16.2±53.2 | 5.90±32.8 ^ab^ | 20.2±62.5 ^f^ | 7.40±42.8 ^e^ | 13.9±49.9 |
| Cheeses | | 2.30±11.1 ^c^ | 2.10±8.80 ^c^ | 7.20±22.1 ^abd^ | 0.80±4.60 ^c^ | 2.64±13.0 | 4.04±15.5 | 3.31±15.1 |
| Eggs and eggs dishes | | 3.60±22.1 ^c^ | 2.70±16.1 ^c^ | 11.4±46.4 ^abd^ | 0.80±8.30 ^c^ | 2.72±16.1 ^f^ | 7.43±35.8 ^eg^ | 3.06±21.3 ^f^ |
| Biscuits, cakes and pastries | | 7.00±24.0 ^d^ | 6.70±24.2 ^d^ | 6.60±26.5 ^d^ | 0.70±6.59 ^abc^ | 10.4±30.2 ^fg^ | 4.87±20.9 ^e^ | 4.74±18.7 ^e^ |
| Cream, ice-cream and dessert | | 11.2±40.9 ^d^ | 10.4±44.7 ^d^ | 5.80±34.1 | 0.30±4.50 ^ab^ | 20.4±54.5 ^fg^ | 7.40±40.3 ^e^ | 11.0±36.0 ^e^ |
| Soups and sauces | | 25.6±65.5 ^d^ | 30.6±65.5 ^d^ | 34.6±100 ^d^ | 0.60±3.80 ^abc^ | 28.9±60.9 ^f^ | 16.5±50.0 ^eg^ | 28.4±65.2 ^f^ |
| Sugar, confectionary, snacks | | 3.08±11.1 ^d^ | 2.77±8.60 ^c^ | 4.61±13.1 ^bd^ | 0.53±3.80 ^ac^ | 2.67±9.67 ^f^ | 5.18±23.5 ^eg^ | 2.84±12.9 ^f^ |
| Breads and rolls | | 11.5±35.5 ^c^ | 11.9±33.4 ^c^ | 35.9±55.5 ^abd^ | 7.10±27.5 ^c^ | 13.0±34.7 ^f^ | 21.3±41.9 ^eg^ | 13.6±36.3 ^f^ |
| Beverages (incl. water) | | 316±345 ^bd^ | 378±424 ^acd^ | 307±379 ^bd^ | 72.0±205 ^abc^ | 406±563 ^f^ | 315±509 ^e^ | 386±595 |

^1^ Values are mean (±SD). ANOVA with Bonferroni correction was applied. Multiple comparison was performed for Classes 1-4 and Classes 5-7 separately. Classes definition: Weekdays: Class 1—88% cereal or toast for breakfast, 23% skip light meal / 28% MF sandwich/ 22% other for light meal, 72% protein and carbohydrates based for main meal; Class 2—64% cereal or toast for breakfast, 23% MFD sandwich / 35% MF sandwich for light meal, 23% skip main meal / 60% protein and carbohydrates based for main meal; Class 3—13% cereal or toast / 44% cooked breakfast / 25% other for breakfast, 57% skip light meal, 64% protein and carbohydrates based for main meal; Class 4—33% cereal and toast / 27% fruit / 37% other, 26% soups and salads / 25 other, 39% protein and carbohydrates based for main meal. ^a^ significant difference from Class 1 (*p* < 0.05); ^b^ significant difference from Class 2 (*p* < 0.001); ^c^ significant difference from Class 3 (*p* < 0.001); ^d^ significant difference from Class 4 (*p* < 0.001). Weekends: Class 5—88% cereal or toast for breakfast, 35% skip light meal / 20% MF sandwich/ 20% other for light meal, 85% protein and carbohydrates based for main meal; Class 6—70% cereal or toast for breakfast 24% MF sandwich / 22% other for light meal, 39% protein and carbohydrates based for main meal; Class 7—45% cooked breakfast, 69% skip light meal, 76% protein and carbohydrates based for main meal. ^e^ significant difference from Class 5 (*p* < 0.05); ^f^ significant difference from Class 6 (*p* < 0.001); ^g^ significant difference from Class 7 (*p* < 0.001).

**Supplemental Table 8.** Intakes^1^ (g/day or ml/day) of selected foods by latent classes during snacking occasions.

|  | Weekdays | | | | Weekends | | |
| --- | --- | --- | --- | --- | --- | --- | --- |
| **Latent Classes** | **Class 1** | **Class 2** | **Class 3** | **Class 4** | **Class 5** | **Class 6** | **Class 7** |
| ***N* (%)** | **2288 (60.0)** | **988 (25.9)** | **360 (9.4)** | **179 (4.7)** | **1249 (57.2)** | **469 (21.5)** | **467 (21.4)** |
| Potatoes/potato dishes | 1.15±12.9 ^cd^ | 2.60±22.9 ^d^ | 5.46±35.6 ^ad^ | 11.9±55.4 ^abc^ | 2.96±27.5 ^g^ | 7.17±40.3 | 11.2±55.0 ^e^ |
| Fruits/fruit dishes | 46.1±95.4 | 44.9±106 | 51.1±122 | 57.1±116 | 38.9±90.9 ^g^ | 34.8±90.1 | 24.4±80.1 ^e^ |
| Milk and yogurt | 49.4±95.2 ^d^ | 57.7±110 | 59.3±122 | 75.0±135 ^a^ | 43.1±85.8 | 47.4±103 | 45.9±114 |
| Meat and meat products | 5.68±28.4 ^bcd^ | 11.7±44.9 ^a^ | 13.6±43.2 ^a^ | 16.9±61.7 ^a^ | 7.67±32.4 ^g^ | 11.9±46.0 | 15.4±52.5 ^e^ |
| Biscuits, cakes and pastries | 14.1±31.2 | 16.8±37.4 | 16.6±37.9 | 17.6±42.2 | 15.3±36.3 | 15.2±42.7 | 15.5±37.9 |
| Cream, ice-cream and dessert | 5.21±30.9 ^d^ | 4.59±24.9 ^d^ | 5.30±24.9 | 12.0±50.1 ^ab^ | 7.47±33.5 ^f^ | 3.37±21.0 ^e^ | 5.59±28.0 |
| Soups, sauces, & miscellaneous | 3.52±29.5 | 5.37±32.7 | 6.87±50.3 | 3.25±17.4 | 3.35±27.3 | 5.82±43.8 | 4.68±36.1 |
| Sugar, confectionary, savoury snacks | 12.4±26.7 ^bcd^ | 17.1±32.0 ^a^ | 16.9±33.9 ^a^ | 22.2±39.1 ^a^ | 13.5±31.7 ^g^ | 14.8±31.1 | 19.0±35.0 ^e^ |
| Breads and rolls | 17.9±38.6 ^bcd^ | 28.8±54.9 ^a^ | 26.5±54.1 ^a^ | 30.9±58.3 ^a^ | 17.4±39.2 | 13.2±35.5 | 17.3±38.4 |
| Beverages (incl. water) | 316±422 ^bd^ | 364±457 ^a^ | 370±499 | 439±489 ^a^ | 334±500 | 308±491 | 357±513 |

^1^ Values are mean (±SD). ANOVA with Bonferroni correction was applied. Multiple comparison was performed for Classes 1-4 and Classes 5-7 separately. Classes definition: Weekdays: Class 1—88% cereal or toast for breakfast, 23% skip light meal / 28% MF sandwich/ 22% other for light meal, 72% protein and carbohydrates based for main meal; Class 2—64% cereal or toast for breakfast, 23% MFD sandwich / 35% MF sandwich for light meal, 23% skip main meal / 60% protein and carbohydrates based for main meal; Class 3—13% cereal or toast / 44% cooked breakfast / 25% other for breakfast, 57% skip light meal, 64% protein and carbohydrates based for main meal; Class 4—33% cereal and toast / 27% fruit / 37% other, 26% soups and salads / 25 other, 39% protein and carbohydrates based for main meal. ^a^ significant difference from Class 1 (*p* < 0.05); ^b^ significant difference from Class 2 (*p* < 0.001); ^c^ significant difference from Class 3 (*p* < 0.001); ^d^ significant difference from Class 4 (*p* < 0.001). Weekends: Class 5—88% cereal or toast for breakfast, 35% skip light meal / 20% MF sandwich/ 20% other for light meal, 85% protein and carbohydrates based for main meal; Class 6—70% cereal or toast for breakfast 24% MF sandwich / 22% other for light meal, 39% protein and carbohydrates based for main meal; Class 7—45% cooked breakfast, 69% skip light meal, 76% protein and carbohydrates based for main meal. ^e^ significant difference from Class 5 (*p* < 0.05); ^f^ significant difference from Class 6 (*p* < 0.001); ^g^ significant difference from Class 7 (*p* < 0.001).

**Supplemental Table 9.** Association between most dominant latent classes computed over 4 days records (weekdays and weekends) and serum ferritin and DBP.

|  | Class 1 weekdays / Class 5 weekends  (*n* = 493) | Class 1 weekdays/  Class 6 weekends  (*n* = 114) | Class 1 weekdays / Class 7 weekends  (*n* = 87) | Class 2 weekdays / Class 5 weekends  (*n* = 100) |
| --- | --- | --- | --- | --- |
|  | Adjusted OR (95% CI) | | |  |
| Increased serum ferritin ^a^ |  |  |  |  |
| Model 1 | 1 (ref) | 1.25 (0.67; 2.33) | 2.75 (1.47; 5.16) ^†^ | 2.11 (1.14; 3.92) * |
| Model 2 | 1 (ref) | 1.48 (0.78; 2.79) | 3.14 (1.63; 6.03) ^†^ | 1.91 (1.00; 3.65) |
| Hypertension |  |  |  |  |
| Model 1 | 1 (ref) | 0.32 (0.09; 1.11) | 1.31 (0.53; 3.21) | 0.86 (0.36; 2.09) |
| Model 2 | 1 (ref) | 0.36 (0.10; 1.26) | 1.46 (0.59; 3.63) | 0.60 (0.22; 1.68) |

* *p* < 0.05, † *p* < 0.01, †† *p* < 0.0001. Classes definition: Weekdays: Class 1—88% cereal or toast for breakfast, 23% skip light meal / 28% MF sandwich/ 22% other for light meal, 72% protein and carbohydrates based for main meal. Weekends: Class 5—88% cereal or toast for breakfast, 35% skip light meal / 20% MF sandwich/ 20% other for light meal, 85% protein and carbohydrates based for main meal; Class 6—70% cereal or toast for breakfast, 24% MF sandwich / 22% other for light meal, 39% protein and carbohydrates based for main meal; Class 7—45% cooked breakfast, 69% skip light meal, 76% protein and carbohydrates based for main meal. Models were adjusted as follows: Model 1: age, sex. Model 2: social class, energy intake. ^a^ Model 1 was only adjusted for age.
